# Genome-Wide Analyses Reveal Genes Subject to Positive Selection in *Pasteurella multocida*

**DOI:** 10.3389/fmicb.2017.00961

**Published:** 2017-05-30

**Authors:** Peili Cao, Dongchun Guo, Jiasen Liu, Qian Jiang, Zhuofei Xu, Liandong Qu

**Affiliations:** ^1^State Key Laboratory of Veterinary Biotechnology, Harbin Veterinary Research Institute, Chinese Academy of Agricultural SciencesHarbin, China; ^2^State Key Laboratory of Agricultural Microbiology, College of Veterinary Medicine, Huazhong Agricultural UniversityWuhan, China

**Keywords:** *P. multocida*, comparative genome analysis, virulence, genetic diversity, positive Darwinian selection

## Abstract

*Pasteurella multocida*, a Gram-negative opportunistic pathogen, has led to a broad range of diseases in mammals and birds, including fowl cholera in poultry, pneumonia and atrophic rhinitis in swine and rabbit, hemorrhagic septicemia in cattle, and bite infections in humans. In order to better interpret the genetic diversity and adaptation evolution of this pathogen, seven genomes of *P. multocida* strains isolated from fowls, rabbit and pigs were determined by using high-throughput sequencing approach. Together with publicly available *P. multocida* genomes, evolutionary features were systematically analyzed in this study. Clustering of 70,565 protein-coding genes showed that the pangenome of 33 *P. multocida* strains was composed of 1,602 core genes, 1,364 dispensable genes, and 1,070 strain-specific genes. Of these, we identified a full spectrum of genes related to virulence factors and revealed genetic diversity of these potential virulence markers across *P. multocida* strains, e.g., *bcbAB, fcbC, lipA, bexDCA, ctrCD, lgtA, lgtC, lic2A* involved in biogenesis of surface polysaccharides, *hsf* encoding autotransporter adhesin, and *fhaB* encoding filamentous haemagglutinin. Furthermore, based on genome-wide positive selection scanning, a total of 35 genes were subject to strong selection pressure. Extensive analyses of protein subcellular location indicated that membrane-associated genes were highly abundant among all positively selected genes. The detected amino acid sites undergoing adaptive selection were preferably located in extracellular space, perhaps associated with bacterial evasion of host immune responses. Our findings shed more light on conservation and distribution of virulence-associated genes across *P. multocida* strains. Meanwhile, this study provides a genetic context for future researches on the mechanism of adaptive evolution in *P. multocida*.

## Introduction

*Pasteurella multocida*, a Gram-negative rod-shaped bacterium, is the type species of the genus *Pasteurella* (Christensen and Bisgaard, 2006). *Pasteurella multocida* is an opportunistic pathogen that can cause multihost diseases, characterized by fowl cholera in poultry, pneumonia and atrophic rhinitis in swine, hemorrhagic septicemia in cattle and rabbit, and bite infections in humans (Boyce et al., 2010). These diseases have led to huge economic losses to the livestock and poultry industry worldwide (Ghaffar and Tariq, 2016). Based on differences in capsular antigens, *P. multocida* is classified into five capsular serogroups A, B, D, E, and F (Carter, 1955). To date, a number of *P. multocida* virulence factors have been extensively studied, including capsule, lipopolysaccharides (LPSs), *P. multocida* toxin, surface adhesins and iron acquisition proteins (Harper et al., 2006).

To explore genetic content of virulence genes in *P. multocida*, the first complete genome sequence of the avian strain Pm70 has been reported in 2001 (May et al., 2001). The research on comparative genomics of *P. multocida* is subsequently fueled by rapid development of next-generation sequencing technology during the last decade. More epidemic strains of *P. multocida* have been whole-genome sequenced to investigate the associations of pathogenesis with the underlying genetic diversity among *P. multocida* genomes (Michael et al., 2012; Abrahante et al., 2014). Several studies on comparative genome analyses have been reported for this pathogen. Analyses of nine *P. multocida* genomes have indicated that there is no clear correlation between phylogenetic relatedness and host predilection or disease (Boyce et al., 2012). In addition, genome-wide comparison of three avian *P. multocida* strains has identified 336 unique genes present in both virulent strains P1059 and X73 but absent in the avirulent strain Pm70, some genes of which may be associated with enhanced virulence or fitness (Johnson et al., 2013). Using genome sequences of 11 haemorrhagic septicaemia (HS) strains and four other *P. multocida* strains, a recent study by Moustafa et al. (2015) has revealed 96 HS-specific genes which could promote the development of disease-specific diagnostic tests.

Except for large genome rearrangement and gene gain or lost, genetic variations referring to single nucleotide polymorphisms (SNPs) and insertions/deletions on the conserved genetic elements also play crucial roles on bacterial virulence, population diversification and adaptation to host niches (Toft and Andersson, 2010). Analyses of SNPs in three *P. multocida* genomes has indicated a higher *d*_N_/*d*_S_ ratio is prone to occur on the genes encoding outer membrane proteins interacted with the host immune system (Johnson et al., 2013). The massive accumulation of genomic sequence data enables researchers to apply more robust *d*_N_/*d*_S_-based methods to trace evolutionary trajectories on the protein-coding genes under positive Darwinian selection exerted by the host immune system. Genome-scale positive selection scanning has been widely used to detect bacterial genes important for host adaptation in many pathogenic taxa, such as *Escherichia coli* (Chen et al., 2006; Petersen et al., 2007), *Campylobacter* (Lefébure and Stanhope, 2009), *Streptococcus* (Lefébure and Stanhope, 2007), *Mycobacterium tuberculosis* (Hongo et al., 2015), and *Actinobacillus pleuropneumoniae* (Xu et al., 2011a). To date, systematical analysis of positive selection on *P. multocida* protein-coding genome is still lacking.

In the present study, we sequenced the genomes of seven *P. multocida* strains isolated in China. Together with 26 public *P. multocida* genome assemblies, a comparative genome analysis was performed to characterize pangenome structure of this pathogenic bacterium. Genetic content and conservation of virulence genes were comprehensively analyzed. Furthermore, a genome-wide evolutionary analysis was carried out to investigate the effects of positive Darwinian selection acting on protein-coding genome. A number of genes and amino acid sites undergoing intensive positive selection were uncovered, which may be associated with the fitness and immunogenic properties of *P. multocida*. These findings provide a genetic context for future researches on diversity of virulence and adaptation evolution in *P. multocida*.

## Methods

### Bacterial strains

*Pasteurella multocida* strains were cultured on brain heart infusion agar (BD Biosciences, San Jose, CA, USA) containing 5% sterile sheep blood and incubated at 37°C for 24~48 h. The *P. multocida* strains were identified *via kmt1* gene and capsular multiplex PCR analysis using primers previously described by Townsend et al. (2001). The ST-type of these strains has been investigated by multi-locus sequence typing based on seven housekeeping genes (*adk, est, pmi, zwf, mdh, gdh*, and *pgi*) in our recent study (Wang et al., 2016). More information about seven *P. multocida* strains sequenced in this study is shown in Table 1. Total genomic DNA was extracted by using the DNeasy tissue kit (Qiagen).

**Table 1:** Summary of genome assembly and gene calling of sequenced ***P. multocida*** strains.

**Strain**	**Host origin**	**Capsular type**	**ST type**	**Clinical syndrome**	**No. of Contigs**	**Coverage**	**Size (bp)**	**No. of CDS**
C48-1	Chicken	A	ST129	Fowl cholera	15	441×	2,304,775	2,096
Pm72-4	Goose	A	ST129	Fowl cholera	26	528×	2,258,903	2,061
Pm731	Goose	A	ST129	Fowl cholera	14	493×	2,260,244	2,028
C51-2	Rabbit	A	ST129	Pneumonic pasteurellosis	31	504×	2,253,947	2,138
C51-3	Rabbit	A	ST5	Pneumonic pasteurellosis	25	612×	2,265,782	2,067
C51-17	Rabbit	A	ST129	Pneumonic pasteurellosis	15	510×	2,235,993	2,057
C44-1	Pig	B	ST122	Atrophic rhinitis	33	515×	2,234,507	2,055

### Genome sequencing, annotation, and alignment

Whole-genome shotgun sequencing was performed by using Illumina MiSeq platform. Illumina sequencing was done by Majorbio Bio-pharm Technology Co., Ltd, China. Genomic DNA library containing 400~500 bp fragments was constructed by TruSeq™ DNA Sample Prep Kit (Illumina). Sequencing experiments were conducted on an Illumina MiSeq sequencer, giving 250-bp paired-end reads and an average ~515-fold genome coverage per sample. Adaptor sequences and low quality ends per read were trimmed by using SeqPrep (https://github.com/jstjohn/SeqPrep) and Sickle (https://github.com/najoshi/sickle/). High quality reads were retained if satisfying the following criteria: minimum mean quality score of 20; minimum read length of 50 bp. Short reads were *de novo* assembled into contigs using the package SOAPdenovo v2.04 (Li et al., 2010), and contigs less than 500 bp were filtered. The genome assemblies were annotated by using an integrated computational pipeline Prokka v1.12 (Seemann, 2014). Briefly, protein-coding genes, tRNAs and rRNAs were predicted by Prodigal v2.6.2 (Hyatt et al., 2010), Aragorn v1.2.36 (Laslett and Canback, 2004), and RNAmmer v1.2 (Lagesen et al., 2007), respectively. Prophage sequences within the genome assemblies were identified by the PHAST web server (Zhou et al., 2011). Based on sequence similarity searching the genome assemblies against the complete genome sequence of *P. multocida* type strain ATCC 43137 using *blastn* (*E*-value cutoff set to 10^−10^) in the package BLAST 2.2.30+ (Camacho et al., 2009), a circular map of pairwise genome alignment was generated and visualized by the tools BRIG v0.95 (Alikhan et al., 2011) and CGview (Grant et al., 2012). The similarity between bacterial genomes was estimated by calculating the average nucleotide identity (ANI) based on BLAST analysis using JSpecies with default parameters (Goris et al., 2007).

Draft genome assemblies have been deposited in the GenBank database under the accession numbers MANI00000000, MAPP00000000, MAPQ00000000, MAPR00000000, MAPS00000000, MAPT00000000, and MBAG00000000.

### Phylogenetic analysis

To infer the phylogeny of *P. multocida* strains, Parsnp v1.2 was employed to perform whole-genome alignment and variant calling using the following parameters: -x -z 25 -c -C 1000 (Treangen et al., 2014). The genome of *P. multocida* type strain ATCC 43137 was used as reference. The core-genome multiple alignment was composed of locally collinear blocks (>25 bp) detected by Parsnp. The aligned columns with recombination signals were further detected by PhiPack (Bruen and Philippe, 2006) and filtered. A phylogenetic tree was reconstructed based on the final alignment of core-genome SNPs using FastTree2 (Price et al., 2010). The resulting tree of the whole-genome phylogeny was then visualized and annotated with the strain properties by using iTOL v3 (Letunic and Bork, 2011).

### Pangenome analysis

A pangenome analysis was carried out to compare genetic content between *P. multocida* genomes. To keep consistency of analysis procedures, we re-annotated the other collected genome assemblies using Prokka. Proteinortho v5.13 (Lechner et al., 2011) was then adopted to cluster orthologous genes according the criteria: more than 80% identity and 80% coverage of the best blast alignments. The clusters of orthologous genes (OGs) present in all strains were determined as the core genes of *P. multocida*. Distribution of OGs among strains was visualized by using FriPan (https://github.com/drpowell/FriPan). Using *blastp* functional annotation and classification of OGs were performed based on the UniRef50 database (Suzek et al., 2007), the cluster of orthologous groups (COG) database (Tatusov et al., 2000), and the virulence factor database VFDB (Chen et al., 2016). Gene annotation was replenished by the top hit with *E*-value cutoff of 10^−20^. Protein functional domain was predicted using the Pfam-A database v29.0 (Punta et al., 2012). OGs annotated to be virulence genes were further screened using *tblastn* searching against *P. multocida* genomes by the approach of BLAST Score Ratio (BSR) (Sahl et al., 2014). A data matrix consisting of the BSR values for each gene across genomes was obtained. Gene distribution and conservation patterns were visualized by using iTOL (Letunic and Bork, 2011). Biological pathway analysis was performed using BlastKOALA and KEGG database (Kanehisa et al., 2016).

### Detection of recombination

To eliminate the potential effect of recombination on positive selection scanning, intragenic homologous recombination was detected for the set of single-copy core OGs. Multiple sequence alignment for each OG cluster was initially performed with amino acid sequences by using T-Coffee v11 (Notredame et al., 2000). The resulting protein sequence alignments were then converted to the codon alignments for the subsequent analysis. Unreliable alignment regions were further removed using Gblocks v0.91 with the codon mode and the default relaxed settings as defined by Talavera (Talavera and Castresana, 2007). Recombination test was conducted using the stand-alone program Genetic Algorithm Recombination Detection (GARD) implemented by HYPHY v2.2 (Kosakovsky-Pond et al., 2006). Additionally, informative sites within each alignment were detected by the PhiPack package. The sequence alignments of the recombining OGs were then partitioned according to the breakpoints inferred by GARD.

### Positive selection scanning

To detect adaptive evolution in the protein-coding genome of *P. multocida*, the rates of synonymous and non-synonymous substitutions were estimated using site-model of the *codeml* program in the PAML v4.8 package (Yang, 2007). Briefly, a phylogenetic tree for each gene was built using the maximum-likelihood approach implemented by PhyML v3.0 (Guindon and Gascuel, 2003). A general time-reversible (GTR) model of nucleotide substitutions with the estimated gamma distributed rate heterogeneity of four categories (Γ4) and a proportion of invariable sites was used in the tree reconstruction. Based on the resulting tree topology, two site-specific models that allow variable ratios (ω) of non-synonymous (*d*_N_) to synonymous substitutions (*d*_S_) among codons were used in our data set: M1a (NearlyNeutral) and M2a (PositiveSelection). The latter model adds an extra site class for a fraction of positively selected sites with ω > 1, indicating positive (adaptive) selection; whereas models M1a only allows site classes with ω varying between 0 and 1 (Wong et al., 2004). A likelihood ratio test was carried out to infer the occurrence of sites subject to positive selection through comparing M1a against M2a. The likelihood statistic (2Δℓ) was calculated and compared with the critical value from χ^2^ distribution with two degrees of freedom. The Bayes empirical bayes (BEB) method was employed to identify positively selected sites under the likelihood framework (Yang et al., 2005).

### Functional analysis of positively selected genes

PSORTb v3.02 web server (Yu et al., 2010) was employed to predict protein subcellular localization. Protein Transmembrane helices were predicted by TMHMM 2.0 (Krogh et al., 2001). Integral β-barrel outer membrane proteins were screened by using BOMP (Berven et al., 2004) and the topology of transmembrane β-barrel was predicted by BOCTOPUS2 (Hayat et al., 2016). Signal peptide cleavage site was predicted by using SignalP v4.1 server (Petersen et al., 2011). Three dimensional structures of positively selected proteins were modeled using Phyre2 server with the intensive model (Kelley and Sternberg, 2015). The amino acid residues subject to positively selective pressure were mapped onto the structure and visualized by Chimera v1.11 (Pettersen et al., 2004).

### Statistical analyses

Multiple testing corrections were performed to control the Type I errors. For the analyses of homologous recombination, recombination breakpoints were inferred by the Shimodaira-Hasegawa test (Shimodaira and Hasegawa, 1999) with Bonferroni-corrected *p*-value and the threshold for significance was set at 0.05. For all genes tested for positive selection, the false discovery rate (FDR) was controlled by using the BY method (Benjamini and Yekutieli, 2001) and the significance level was set to 10%. Chi-square test and Fisher-exact test where appropriate were used to assess associations for the gene count data in the individual COG categories; Bonferroni corrections for multiple tests were applied. All computational analyses were carried out using *in-house* Perl scripts and R 3.2.1 (R Core Team, 2015).

## Results

### General features of newly sequenced genomes

In the present study, seven *P. multocida* strains were chosen for whole genome shotgun sequencing, including three strains C51-2, C51-3, C51-17 isolated from rabbit, two strains Pm72-4 and Pm731 isolated from goose, strain C48-1 isolated from chicken, and strain C44-1 isolated from pig (Table 1). After genome assembly, the average number of contigs per genome was 23 with a range from 14 to 33. The depth of genome coverage was 441- to 612-fold (Table 1). The average GC content of each genome was 40.3%, which was consistent with that of a complete *P. multocida* chromosome (Liu et al., 2012). The median number of coding sequences (CDSs) per strain was 2,061, the largest number was 2,138 for strain C51-2, and the smallest was 2,028 for strain Pm731. Additionally, the newly sequenced strains possessed pairwise ANI values of 98.06~99.96% compared with eight representative *P. multocida* strains (Table S1). The high ANI values of pairwise genome comparisons again confirmed these strains belonging to *P. multocida*. All genome assemblies of seven strains have been deposited in the GenBank database.

**Figure 1:**
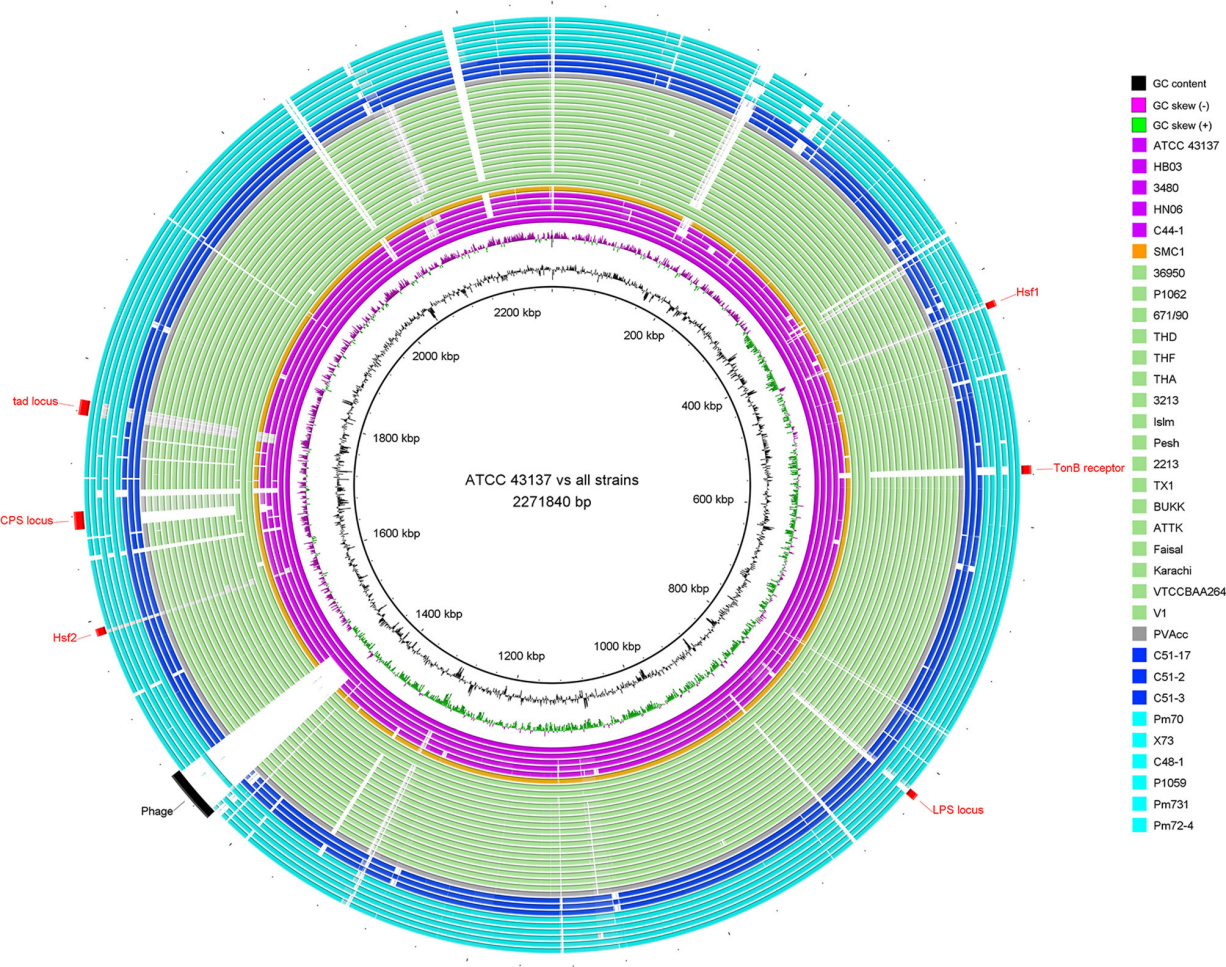
Circular diagram of genomic sequence conservation of 33 *P. multocida* strains. Rings are numbered from 1 (outermost ring) to 35 (innermost ring). The outermost 33 rings show pairwise nucleotide alignment between 32 genome assemblies of *P. multocida* and the reference genome of *P. multocida* type strain ATCC 43137. Each ring is color-coded according to host origins of isolates: magenta for swine source strains, light green for bovine source strains, blue for rabbit source strains, cyan for avian source strains, orange for human source strain, and gray for strain PVAcc without host metadata. BLAST matches (minimum sequence identity of 80% and *E*-value cutoff of 10^−10^) are colored from darkest to lightest shade. The innermost ring shows mean centered GC content (outward black bar: above mean; inward bar: below mean) of the ATCC 43137 genome. The second innermost ring shows GC skew plot [(G-C)/(G+C); green indicates ratio > 0 and purple indicates ratio < 0]. Red labels and arcs denote genomic regions encoding several known virulence factors of *P. multocida*; black label and arc denote a prophage region predicted by PHAST (Zhou et al., 2011).

For a glimpse of genome similarity of *P. multocida* strains, pairwise sequence alignments between genomes of *P. multocida* reference strain ATCC 43137 and other strains is shown in Figure 1. We found the majority of genomic regions between the reference strain ATCC 43137 and the other *P. multocida* strains were highly conserved. Among seven newly sequenced *P. multocida* strains in our study, the proportion of all matched sequences (>95% identity) accounting for the ATCC 43137 genome (2,271,840 bp) was ranged from ~89.5% (C44-1 vs. ATCC 43137) to ~92.7% (C48-1 vs. ATCC 43137). Genomic fragments (>200 bp) that are present in the ATCC 43137 genome but absent in the newly sequenced genomes were summarized and shown in Table S2. In accordance with the type-A capsule of *P. multocida* strain ATCC 43137 (Davenport et al., 2014), the biosynthetic loci of capsular polysaccharide (CPS) were highly conserved in the newly sequenced genomes of six strains possessing type-A capsule. Unsurprisingly, the locus of type-A capsule was absent in strain C44-1 and the other strains bearing non-type-A capsule. Except the CPS locus, we also observed genomic islands relative to the other known VFs, including LPS locus, TonB receptor protein, autotransporter surface adhesins Hsf, and *tad* (tight adherence) locus (Figure 1). These particular genomic regions have been depicted by a recent pangenome analysis based on nine *P. multocida* genomes (Boyce et al., 2012). Gene content and conservation of these featured genomic regions were analyzed in details below. In addition, an intact prophage (41.6 kb), which was localized in the vicinity of the replication terminus of the ATCC 43137 genome, was also present in the genomes of three serotype-A strains (HB03, 3480, and X73) but not all. The prophage island contains 58 CDSs, 40 of which encode phage-related proteins.

### Phylogeny of *P. multocida*

**Figure 2:**
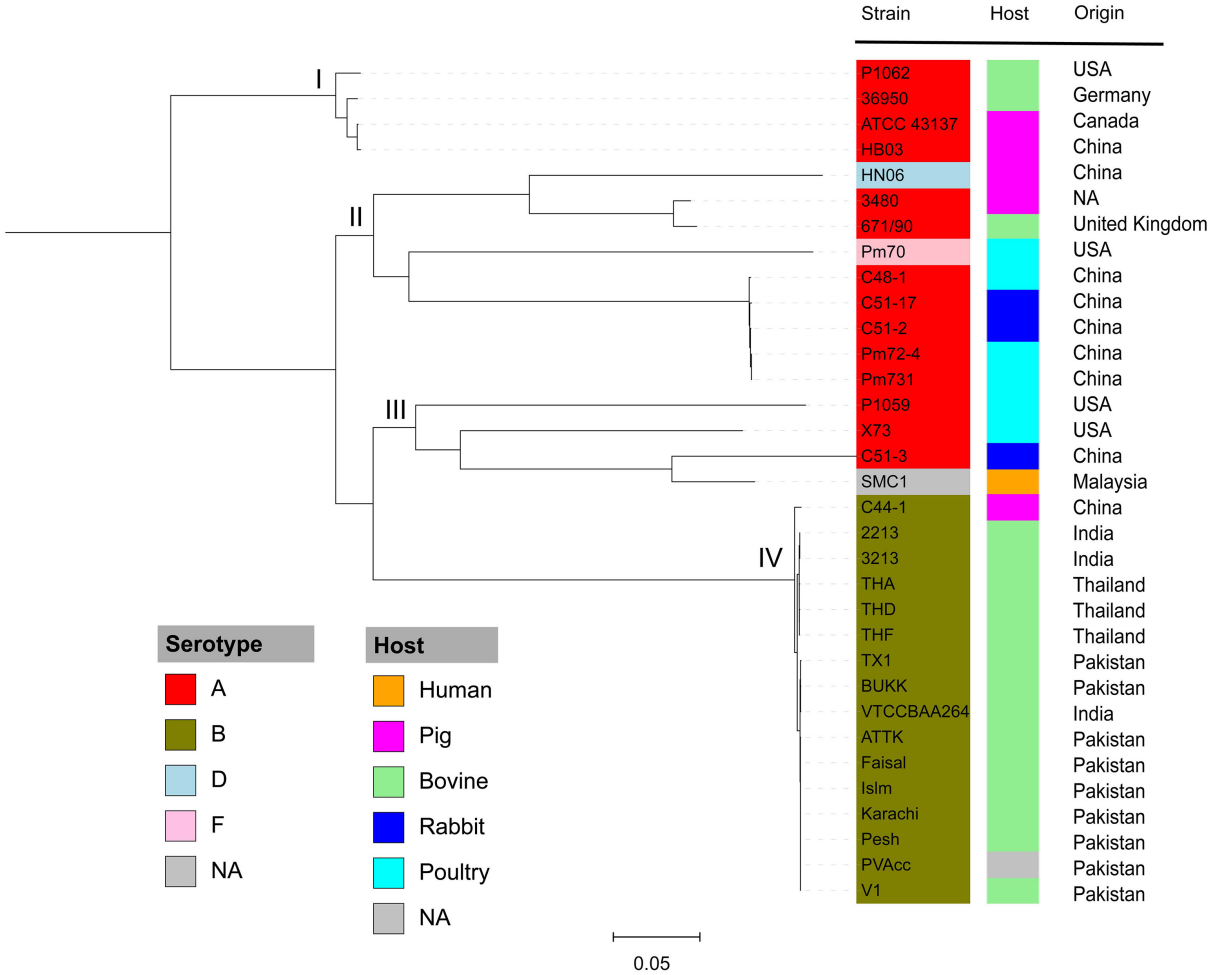
Maximum likelihood phylogeny of *P. multocida*. The phylogenetic tree was reconstructed based on SNPs in the core genomes of *P. multocida* strains using the Parsnp program (Treangen et al., 2014). Phylogenetic group was manually set and designated as groups I, II, III, and IV. All strains are color-coded according to their serotypes A, B, D, and F. The host origin of each strain is denoted by the right color strip with the same scheme as in Figure 1. The geographical location of isolation for each strain is shown. The gray box labeled as NA indicates the metadata is not available for the related strains.

To understand the phylogenetic relationships among *P. multocida* isolates, 26 publicly available *P. multocida* genomes were collected and compared with seven sequenced genomes in this study (Table 2). As shown in Figure 2, a maximum-likelihood phylogeny of *P. multocida* was reconstructed based on SNPs detected in the core-genome of all strains. It was apparent that four major phylogenetic groups were found in the tree. All serotype-B strains, including fifteen bovine source strains and one swine source strain, were clustered into the same phylogenetic group IV. For strains in the phylogenetic group II, we observed that three poultry source stains (C48-1, Pm72-4, and Pm731) and two rabbit source strains (C51-17 and C51-2) were closely related, implicating these strains from both hosts probably derived from a recent common ancestor. Phylogenetic group I containing two swine source strains (ATCC 43137 and HB03) and two bovine source strains (36950 and P1062) was very distantly related to group II and III, in which serotype-A strains were also dominated. In addition, the pathogenic strain SMC1 isolated from an adult female in Malaysia and the virulent strain C51-3 isolated from rabbit in China were assigned to a common clade. Consistently, the ANI value between strains C51-3 and SMC1 was 99.31%, indicating both are closely related strains within *P. multocida*. It was noting that none of obvious associations between phylogenetic groups and phenotypic characteristics of *P. multocida* strains could be presented, perhaps due to the limited genome data collected in this study. This observation is in line with a previous study on comparative genomics analyses of nine *P. multocida* strains (Boyce et al., 2012).

**Table 2:** Public *P. multocida* genome assemblies analyzed in this study.

**Strain name**	**Genome acc. no**.	**Genome status**	**Size (Mbp)**	**Host**	**Capsular type**	**References**
Pm70	AE004439	Complete	2.26	Chicken	F	May et al., 2001
36950	CP003022	Complete	2.35	Bovine	A	Michael et al., 2012
ATCC 43137	CP008918	Complete	2.27	Pig	A	Davenport et al., 2014
HN06	CP003313	Complete	2.40	Pig	D	Liu et al., 2012
HB03	CP003328	Complete	2.31	Pig	A	NA
3480	CP001409	Complete	2.38	Pig	A	NA
X73	AMBP00000000	Draft	2.27	Chicken	A	Johnson et al., 2013
P1059	AMBQ00000000	Draft	2.31	Turkey	A	Johnson et al., 2013
P1062	ASZP00000000	Draft	2.51	Bovine	A	Abrahante et al., 2015
671/90	ARWR00000000	Draft	2.25	Bovine	A	Lainson et al., 2013
SMC1	LNCO00000000	Draft	2.28	Human	NA	Kavousi et al., 2016
V1	JQAI00000000	Draft	2.35	Bovine	B	Moustafa et al., 2015
THD	JQAF00000000	Draft	2.30	Bovine	B	Moustafa et al., 2015
THF	JQAG00000000	Draft	2.30	Bovine	B	Moustafa et al., 2015
THA	JQAE00000000	Draft	2.30	Bovine	B	Moustafa et al., 2015
3213	JNOL00000000	Draft	2.30	Bovine	B	Abrahante et al., 2014
Islm	JQAB00000000	Draft	2.35	Bovine	B	Moustafa et al., 2015
Pesh	JQAC00000000	Draft	2.35	Bovine	B	Moustafa et al., 2015
2213	JNOK00000000	Draft	2.30	Bovine	B	Abrahante et al., 2014
TX1	JQAH00000000	Draft	2.41	Bovine	B	Moustafa et al., 2015
PVAcc	JQAD00000000	Draft	2.35	NA	B	Moustafa et al., 2015
BUKK	JQAO00000000	Draft	2.36	Bovine	B	Moustafa et al., 2015
ATTK	JQEA00000000	Draft	2.35	Bovine	B	Moustafa et al., 2015
Faisal	JQEB00000000	Draft	2.36	Bovine	B	Moustafa et al., 2015
Karachi	JPHI00000000	Draft	2.37	Bovine	B	Moustafa et al., 2015
VTCCBAA264	ALYC00000000	Draft	2.28	Bovine	B	Vaid et al., 2014

*NA, not available*.

### Pangenome of *P. multocida*

**Figure 3:**
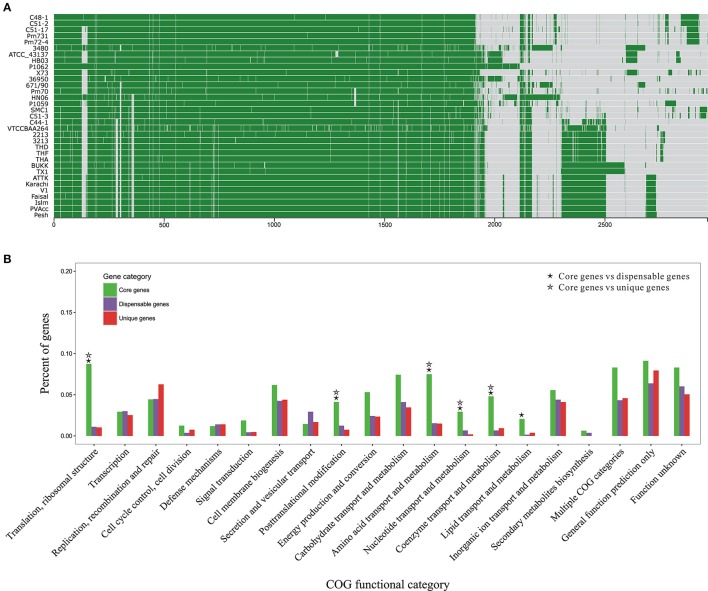
Pan-genome structure and function of *P. multocida*. **(A)** A pan-genome map containing 2,966 OG clusters in all *P. multocida* genomes detected by Proteinortho (Lechner et al., 2011). Each block strands for a gene: green for presence and gray for absence. **(B)** COG-based binning of core genes, dispensable genes, and strain-specific genes. The abscissa denotes different COG functional categories. The ordinate denotes the proportion of genes in each COG category and details are summarized in Table S3. Two COG functional categories (“RNA processing and modification” and “Cell motility”) including only one OG are not displayed, as well as the genes encoding proteins without homologs in the COG collection. Significant enrichment of gene occurrence in the individual category is marked by asterisks (FDR < 0.001; Chi-square test).

To get a glimpse of distribution of protein clusters across *P. multocida* genomes, we performed pangenome analyses based on 70,565 CDSs from all 33 genomes tested in this study (Figure 3A). The total number of *P. multocida* orthologous gene (OG) clusters, also including unique genes specific to individual strain, was 4,036 (Table S3). Of these OGs, 39% were single-copy genes present in all tested strains and constituted the core genome of *P. multocida*, 33% were dispensable genes that were possessed by at least two strains but not all, 27% were unique genes per strain, and the remaining were multiple-copy genes (Figure S1). The dispensable genes together with the unique genes are likely to play crucial roles on strain-specific phenotypes or pathotypes. Genetic content and diversity on these accessory genes across strains were analyzed below.

Functional classification of gene repertoire in the pangenome of *P. multocida* is shown in Figure 3B and details for COG-based binning are summarized in Table S4. The distribution of genes assigned to the major COG functional categories was remarkably different between core genes and dispensable/unique genes. In comparison to the dispensable and unique genes, the core genes encoding proteins involved in the fundamental metabolic activities were significantly abundant in the following COG categories: “Translation, ribosomal structure,” “Posttranslational modification,” “Amino acid metabolism,” “Nucleotide metabolism,” “Coenzyme metabolism” (FDR < 0.001; Chi-square test) (Figure 3B). Enrichment of genes involved in “Lipid metabolism” was also observed for the comparisons between core and dispensable genes (FDR < 0.001), and between core and unique genes (*p*-value < 0.001 and FDR = 0.13), respectively. Additionally, gene products of both categories “Carbohydrate metabolism” and “Energy metabolism” were more abundant in the set of core genes with less stringent FDR < 0.01 (Table S4). The core genes present in these functional categories, especially translation-associated genes, are essential for bacterial growth and survival. In contrast, genes coding for hypothetical proteins without homologs in the COG collection were accounting for about half of the dispensable genes and strain-specific genes, respectively (Table S4).

### Gene patterns of virulence factors

**Figure 4:**
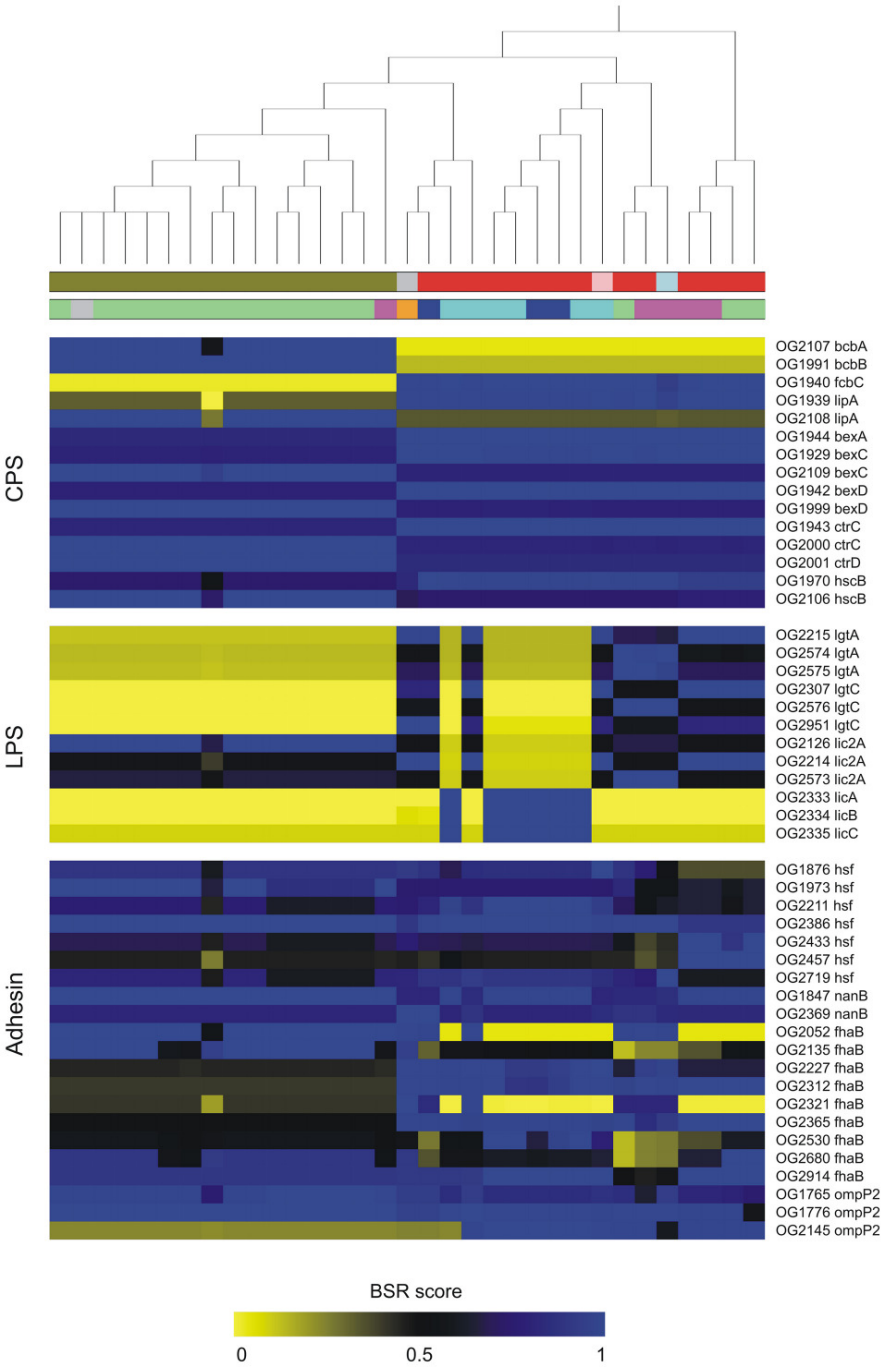
Distribution and conservation of selected genes coding for putative virulence factors in *P. multocida*. Putative virulence-associated genes were identified by *blastp* searching OGs against VFDB. The conservation of virulence markers in the pan-genome across all genomes is color-coded based on BLAST Score Ratio (BSR) values. The top dendrogram is the core SNP phylogeny. Color strips underneath the dendrogram represent serotypes and host origin with the same scheme shown in Figure 2. Gene annotations are detailed in Table S4.

Based on BLAST searching against the VFDB database, we found 291 OGs encoding putative VFs in the pangenome of *P. multocida*. Details of these OGs together with Pfam functional domains were summarized in Table S5. Among these, 170 are core genes present in all *P. multocida* genomes tested and many of these encode products associated with common VFs. For instance, *P. multocida* LPS consist of lipid A, a core oligosaccharide, and a surface-exposed O-antigen (Christensen and Bisgaard, 2006). As is well known, O-antigen is the most variable component of the LPS, whereas, the structure of lipid A is highly conserved in general (Steimle et al., 2016). In accordance with the LPS structure, genes essential for biosynthesis of lipid A (*lpxA, lpxB, lpxC, lpxD, lpxH, lpxK, htrB, msbB, kdsA, kdsB, kdtA*, and *kdkA*) and core oligosaccharide (*gmhA, rfaE, rfaF*, and *lgtF* and *waaQ*) are uniformly present in all *P. multocida* strains (Table S5). In addition, pili have been observed with *P. multocida* (Christensen and Bisgaard, 2006) and consistently, five genes *pilRQFBG* involved in biogenesis of type IV pili were identified. In comparison to the core genes, accessory genes associated with bacterial virulence could be used as markers to distinguish pathovars (Rasko et al., 2008; Sahl et al., 2014). The distribution and conservation of virulence associated gene markers across *P. multocida* strains is shown in Figure 4 and discussed below.

### Highly conserved genes in *P. multocida*

Among 1,559 single-copy core genes, 124 gene alignments containing few informative sites less than two were identified as highly conserved genes in *P. multocida*. Of the alignments of these conserved genes, 19 had no occurrence of nucleotide substitutions in the individual alignments. Notably, all these evolutionarily conversed genes were significantly abundant in the COG category “Translation” which 37 genes were affiliated to (*p*-value < 0.001; Chi-square test). It also confirms that genes involved in the translation machinery are evolving slowly with few nucleotide substitutions, perhaps due to functional constraints required by the fundamental cell cycle and bacterial survival (Jordan et al., 2002; Xu et al., 2011a).

### Recombination of *P. multocida* core genes

Recombination test on the alignments of each single-copy core genes showed 7% (107 genes) were identified to exhibit significant evidence for homologous recombination in the individual alignments (FDR < 5%) (Table S6). Most of these genes had one recombinant breakpoint except for five genes (i.e., *his7, his81, y112, dauA*, and *pstC*) with multiple breakpoints. In comparison, Soyer reported that approximately 8% (270 of the 3,316 genes analyzed) of core genome genes present in five *Salmonella* genomes showed evidence for intragenic recombination (Soyer et al., 2009). Additionally, analysis of four *E. coli* and two *Shigella* genomes found 6% of core genes exhibited evidence for recombination (Petersen et al., 2007). Among the recombining genes in *P. multocida*, 13 are coding for putative virulence-associated proteins (Table S6). Besides, two genes (*dnaB*, replicative DNA helicase; *glyS*, Glycine–tRNA ligase beta subunit) have been experimentally confirmed to be preferentially expressed during acute *P. multocida* infection by selective capture of transcribed sequences (Guo et al., 2012).

### Evidence for 35 *P. multocida* genes subject to positive selection

To detect potential genes of pathogenic *P. multocida* in response to host niches, positive selection analysis was performed on the alignments of non-recombinant genes/fragments using codeml-site-model with the nested models M1a/M2a, followed by multiple tests (FDR < 10%). A total of 35 genes were identified to be subject to strong selection pressure (Table 3). It was obvious that the genes were more abundant in the COG categories “Cell membrane biogenesis,” “General function prediction only,” “Function unknown,” and “Not in COG” (Table S7). Furthermore, based on the analyses of protein subcellular location, nearly half (*n* = 16) of all positively selected genes are coding for products localized on cell surface/membrane (Table 3). The enrichment of membrane-associated genes again supports a previous notion that positive selection driven by host immune and defense systems acts on surface/membrane structures in many pathogens (Fitzpatrick et al., 2005; Chen et al., 2006; Johnson et al., 2013). Some of the membrane-associated proteins have been implicated as crucial VFs associated with bacterial colonization and adaptation to host niches (Lin et al., 2002).

**Table 3:** *P. multocida* genes that show evidence for positive Darwinian selection.

**Symbol**	**OG ID^a^**	**2 Δln*L*^b^**	***p*^c^**	**ω^d^**	**Localization^e^**	**Function of coding product**
*recB*	OG0010	29.96	0.014	10.44108	CM	RecBCD enzyme subunit
*yedZ*	OG0031	18.24	0.031	23.82634	CM	Sulfoxide reductase heme-binding subunit
–	OG0056	23.39	0.048	10.60501	CP	Uncharacterized protein
*moaE*	OG0096	41.55	0.135	34.05063	CP	Molybdopterin synthase catalytic subunit
*tehB*	OG0127	18.16	0.007	64.28752	CP	Probable S-adenosyl-L-methionine-dependent methyltransferase
*yfbV*	OG0164	23.53	0.026	94.19119	CM	membrane protein
*ompP5*	OG0218	66.05	0.068	4.7802	OM	Outer membrane protein P5, OmpA family
*pqqL*	OG0232-2	40.09	0.008	20.73989	NA	Probable zinc protease
–	OG0350	18.49	0.017	12.15016	CP	Uncharacterized protein
*ruvB*	OG0371	23.96	0.144	222.90698	NA	Holliday junction DNA helicase B
*rffG*	OG0414	17.52	0.059	9.15245	CP	dTDP-glucose 4,6-dehydratase 2
*tmcA*	OG0427	21.59	0.030	6.88468	CM	tRNA(Met) cytidine acetyltransferase
*ybeY*	OG0429	16.35	0.048	26.31054	CP	Endoribonuclease
–	OG0442	25.64	0.008	65.96138	NA	Uncharacterized protein
*omp47*	OG0453-1	59.07	0.067	8.32257	OM	47 kDa outer membrane protein
*omp47*	OG0453-2	16.99	0.033	5.51695	OM	47 kDa outer membrane protein
*hptE*	OG0503	22.84	0.122	3.76268	CP	Putative 1,6-LD-heptosyltransferase
*gabR*	OG0574	20.52	0.018	16.26975	CP	HTH-type transcriptional regulator
*sdaC*	OG0597	21.79	0.042	9.76688	CM	Serine transporter
*tamB*	OG0789	19.06	0.014	7.32673	CM	Translocation and assembly module
*trmA*	OG0794	24.53	0.013	25.26307	CP	tRNA/tmRNA (uracil-C(5))-methyltransferase
*selB*	OG0816	19.27	0.012	9.73775	CP	Selenocysteine-specific elongation factor
*ftsW*	OG0896	19.68	0.078	4.00107	CM	Putative lipid II flippase
*qseC*	OG0958	17.18	0.003	46.23088	CM	Sensor protein
–	OG1056	28.38	0.059	6.96683	CM	Uncharacterized protein
*int*	OG1057	16.57	0.013	9.45132	CM	Site-specific recombinase
*wzz*	OG1247	25.13	0.033	12.34087	TMHs	Lipopolysaccharide chain length determining protein
*yofA*	OG1284-2	18.78	0.081	217.2752	CP	HTH-type transcriptional regulator
*alsB*	OG1322	54.05	0.070	35.20812	PP	D-allose-binding periplasmic protein
*ntpA*	OG1373	17.04	0.070	7.16529	CP	Non-canonical purine NTP pyrophosphatase
*icsA*	OG1421-2	26.70	0.056	5.75081	OM	Outer membrane protein IcsA autotransporter
*surE*	OG1447	41.57	0.087	8.39148	CP	5′-nucleotidase
*envC*	OG1501	19.46	0.005	28.22338	OM	Murein hydrolase activator
*ftsY*	OG1508	19.81	0.005	15.79486	CP	Signal recognition particle receptor
*betB*	OG1518	25.63	0.004	19.7691	CP	NAD/NADP-dependent betaine aldehyde dehydrogenase
*hgbA*	OG1564	47.13	0.013	10.96349	OM	TonB-dependent hemoglobin/transferrin/lactoferrin receptor family protein

a *Ortholog identifiers were taken from the pangenome analysis that was summarized in Table S2*.

b *2 ΔlnL denote the likelihood ratio*.

c *The proportion of the amino acid sites undergoing positive selection*.

d *ω is equal to the ratio of d_N_ to d_S_ for amino acid sites under positive selection (model M2a)*.

e *Subcellular localization with the abbreviations standing for Cytoplasmic Membrane (CM), Outer Membrane (OM), Transmembrane helices (TMHs), Cytoplasmic (CP), Periplasmic (PP), Unknown (NA)*.

## Discussion

In this report, we performed deep genome sequencing for seven *P. multocida* strains. Together with 26 publicly available and high quality genome sequences of *P. multocida*, a comprehensive investigation was carried out to study pangenome characterization, genetic diversity of virulence genes, and evolutionary selection forces operating on the protein-coding genome of this zoonotic pathogen.

### Diversity of virulence genes in *P. multocida*

Diversity of bacterial surface polysaccharides is often associated with genetic heterogeneity of the capsule and LPS loci (Howell et al., 2013). In the biosynthetic loci of *P. multocida* capsules, two genes *bcbA* and *bcbB* encoding enzymes that catalyze the conversion of UDP-*N*-acetylglucosamine to *N*-acetyl-D-mannosaminuronic acid (Townsend et al., 2001), were found to be serotype-B specific (Figure 4). In contrast, another capsule biosynthesis gene *fcbC* encoding UDP-glucose dehydrogenase is present in all strains except for the serotype-B strains (Figure 4). Two variants of *lipA* (OG1939, 696 aa; OG2108, 682 aa) encoding CPS modification protein are specific to serotype-B and non-serotype-B strains, respectively. Both products of *lipA* share 54% amino acid (aa) sequence identity (74% similarity) and 41% identity (61% similarity) with a LipA homolog from *Neisseria meningitidis* MC58, which is involved in the phospholipid modification of the CPS and its translocation to the cell surface (Frosch and Müller, 1993). In addition, it was apparent that five genes *bexDCA* and *ctrCD* involved in ATP-driven capsule export and gene *hcs* involved in post-polymerization (Satola et al., 2003) showed particular patterns of sequence conservation, which could be used to distinguish strains of serotype-B and non-serotype-B (Figure 4). On the other hand, glycosyltransferases participating in LPS biosynthesis have been considered to be serotype specific (Davies et al., 2013). Glycosyltransferases encoded by three accessary genes *lgtA, lgtC*, and *lic2A* were identified in the pangenome of *P. multocida*. Variants of both *lgtA* and *lgtC* are absent in all serotype-B strains and six serotype-A strains. Notably, three genes (OG2333–2335) that are present in the six type-A strains mentioned above share 41, 36, and 47% aa sequence identity, respectively, with the proteins LicA, LicB, and LicC encoded in the *Haemophilus influenza lic* operon responsible for the addition of phosphorylcholine to LPS (Humphries and High, 2002).

A dozen of genes coding for autotransporter (AT) proteins of the type V secretion pathway were identified in the pangenome of *P. multocida* (Figure 4), which are key players participating in virulence and pathogen-host interactions (Kline et al., 2009). Six variants of *hsf* encoding trimeric AT adhesins (TAAs) were found in the pangenome, all of which carry at least two of three typical TAA structure domains including an N-terminal head (PF05658), a stalk domain (PF05658), and a C-terminal membrane anchor (PF03895) (Table S5) (Mikula et al., 2012). Furthermore, two variants (OG1847, 1055 aa; OG2369, 1063 aa) of *nanB* encoding sialidase with 2-6′ and 2-3′ sialyl lactose specificity are present in the pangenome, which may contribute bacterial colonization on mucosal surfaces (Mizan et al., 2000). Both sialidases possess a signal peptide mediating protein transportation across the inner membrane and a C-terminal autotransporter domain (PF03797) responsible for transportation of the BNR repeat-like domain (PF13088) across the outer membrane (Leo et al., 2012). In addition, the protein products of nine *fhaB* variants are homologous to the *Bordetella pertussis* filamentous haemagglutinin (FHA), which is a surface-exposed protein functioning as both a primary adhesion and an immunomodulator (Inatsuka et al., 2005). It was worth nothing that the majority of these FHA-like proteins harbor an extended signal peptide of Type V secretion system (PF13018), a haemagglutination activity domain (PF05860) followed by the haemagluttinin repeats (PF13332) (Table S5). Mosaic patterns of sequence conservation revealed remarkable diversity of these virulence genes that could be used as genetic markers across the population of *P. multocida*. Detection of variants of virulence genes will facilitate the PCR-based epidemiological study to elucidate the associations between genotypes and phenotypes of *P. multocida* strains (Tang et al., 2009).

### Positively selected genes in *P. multocida*

**Figure 5:**
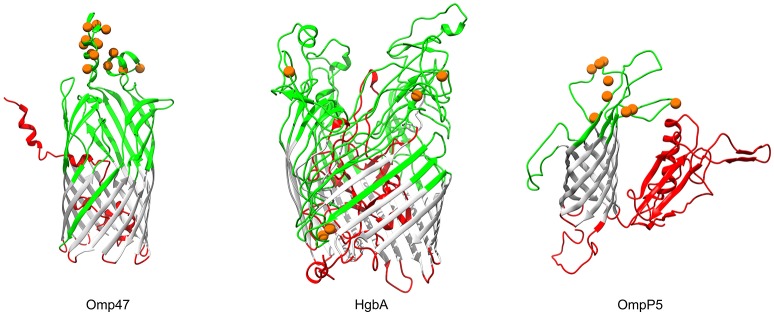
Three-dimensional structural models of β-barrel outer membrane proteins. Topology of three outer membrane proteins undergoing positive selection are color-coded according to the BOCTOPUS2 predictions (Hayat et al., 2016): red for inner-loops, gray for transmembrane β-strands, and green for outer-loops. Orange spheres stand for amino acid residues that are subject to strong positive selection (posterior probability > 95%).

In Gram-negative bacteria, β-barrel outer membrane proteins are porins that mainly function in passive nutrient intake and active ion transport (Berven et al., 2004), as well as dynamic interactions with the host immune system (Massari et al., 2003). Bacterial transmembrane (TM) β-barrels consist of an even number of anti-parallel β-strands, generally 8–22 TM β-strands (Tamm et al., 2001). In our analysis, products of three genes (OG0453, *omp47*, 47 kDa outer membrane protein; OG1564, *hgbA*, TonB-dependent receptor family protein; OG0218, *ompP5*, outer membrane protein P5), which were predicted to be transmembrane β-barrel porins, showed strong evidence for positive selection (FDR < 10%; Table 3). The number of TM-strands in Omp47, HgbA, OmpP5 is 14, 20, and 8, respectively (Figure 5). The results of the BEB analyses showed that 14 amino acid residues of *P. multocida* Omp47 were subject to intensively positive selection (Table S7). Interestingly, all detected residues are located on the third and fourth extracellular loops, which are likely to be potential antigenic epitope. Omp47 is an adhesin binding to the host fibronectin and it has been experimentally validated to be immunogenic (Wheeler, 2009; Prasannavadhana et al., 2014). Genetic alterations on the extracellular region of Omp47 may be positively selected by recognition of fibronectin isoforms in different host niches. Notably, recombination signal was also observed in the alignment of *omp47* (1,323 nt), with one breakpoint detected at position 651 (Table S6). Another β-barrrel porin, HgbA (971 aa) of *P. multocida* shares TonB dependent receptor family (PF00593) at the C-terminus and Plug domain (PF07715) at the N-terminus with *H. influenza* hemoglobin-haptoglobin binding protein HhuA (1025 aa) that is mostly involved in heme and iron acquisition (Maciver et al., 1996). Positive selection acting on *hgbA* may enable this pathogen to better scavenge host iron-binding complexes in various niches. OmpP5 is a major structural protein present in many gram-negative bacteria (Davies and Lee, 2004) and it has both immunodominant and host-adhesive domains (Novotny et al., 2000). Significant evidence for intensively positive selection on *ompP5* was detected (FDR = 5.5e-11) and nine positively selected sites were all located in the extracellular space (Figure 5), perhaps associated with bacterial evasion of host immune responses. *Pasteurella multocida* OmpP5 (353 aa) shares high sequence similarity with the homologs outer membrane protein P5 (353 aa, 64% identity) of *H. influenza* and outer membrane protein A (OmpA, 346 aa, 47% identity) of *E. coli*. Similarly, a previous study has reported that *E. coli* OmpA that is a prime target of the host immune system during infection shows strong evidence of positive selection on the extracellular loops (Petersen et al., 2007).

**Figure 6:**
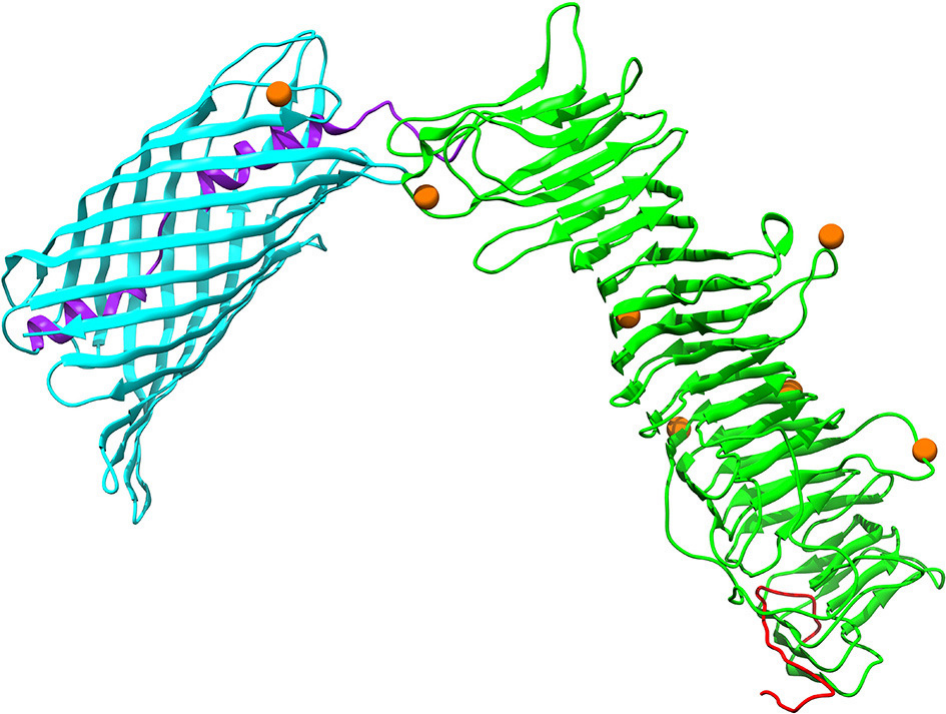
Three-dimensional structural models of the autotransporter protein IcsA. Protein secondary structure is color-coded based on the predicted PFAM domains and signal peptide cleavage site as well as modular organization previously described by Nicolay et al. (2015): an N-terminal signal peptide in red, a secreted mature passenger domain in green, and a C-terminal translocator domain in cyan with an upstream α-helical linker in purple. Orange spheres stand for amino acid residues that are subject to strong positive selection (posterior probability > 95%).

Autotransporter proteins are typically VFs in *P. multocida* (Nicolay et al., 2015). The gene *icsA* (OG1421) encoding an autotransporter related to the Type V secretion system, was undergoing significantly positive selection (FDR = 0.002) (Table S7). It was worth noting that intragenic homologous recombination also occurred on *icsA* (2,556 nt), with one breakpoint at position 331 (Table S6). The outer membrane protein IcsA of *P. multocida* harbors a β-barrel domain (PF03797) at the C-terminal followed by an α-helical linker essential for outer membrane translocation of the passenger domain pertactin (PF03212) with characteristic β-helix (Nicolay et al., 2015). Structural mapping of seven positively selected sites on IcsA showed that the majority of sites (i.e., residues 159, 220, 315, 481, and 690) were located at the loops connecting the β-strands (Figure 6). Additionally, *P. multocida* IcsA shares 48% sequence similarity with the *Shigella flexneri* homolog that could interact with the host proteins vinculin and neural Wiskott-Aldrich syndrome protein implicated in the actin-based motility (Purdy et al., 2007).

LPS in the outer membrane of *P. multocida*, which is a protective antigen stimulating humoral immunity, plays a critical role in the pathogenesis of disease (Harper et al., 2006). Three genes (*rffG*, OG0414, dTDP-glucose 4,6-dehydratase 2; *ntpA*, OG1373, non-canonical purine NTP pyrophosphatase; *wzz*, OG1247, LPS chain length determining protein) involved in biogenesis of LPS were subject to positive selection. The products of both genes *ntpA* and *rffG* share 83 and 64% identity with *H. influenza* OrfM and RffG, respectively, which are involved in the formation of bacterial endotoxin (Chen et al., 2016). Besides, the product of the positively selected gene *wzz* is an O-unit-processing enzyme, which was predicted to have two transmembrane helices (TMHs) (Figure S2) typical for the O-antigen chain length determining proteins of Gram-negative bacteria, such as *H. parasuis* and *A. pleuropneumoniae* (Xu et al., 2011b). Notably, three (77, 205, 241) out of four positively selected amino acid residues were located in the extracellular side (positions 36-247) of *P. multocida* Wzz, and the remaining site occurred in the TMH (positions 13-35) at the N-terminus.

The other gene *ftsW* coding for an integral membrane protein (Figure S2) functioning as a transporter of lipid-linked peptidoglycan precursors across the cytoplasmic membrane during cell division (Mohammadi et al., 2011), was also subject to positive selection (FDR = 0.028). Additionally, a functional unknown transmembrane protein encoded by the gene OG1056 (Figure S2) showed significant evidence for intensive positive selection (FDR < 0.001). The above analyses implicated that the adaptive changes is closely associated with the genes involved in the biosynthesis of cell surface/membrane components, perhaps due to interactions with the host immune and defense system.

Intriguingly, three *P. multocida* genes (*recB*, a subunit of RecBCD enzyme; *int*, site-specific recombinase; and *ruvB*, holliday junction DNA helicase B) with functional roles in DNA recombination and repair showed evidence for positive selection (Table 3). Similar phenomena have already been proposed for diversifying selection on the gene products constituting basal DNA repair machinery, such as *recB* in *Neisseria* (Yu et al., 2014), *recC* in *E. coli* (Chen et al., 2006) and basal DNA repair genes in ionizing-radiation-resistant bacteria (Sghaier et al., 2008). In addition, *yedZ* (OG0031) encoding a sulfoxide reductase heme-binding subunit with six TMHs (Figure S2), which was predicted to be an integral component of plasma membrane (GO: 0005887) and involved in protein repair (GO: 0030091), was also undergoing strong selection pressure. The precise function of these adaptive evolving genes associated with the metabolism of protein and DNA, to our knowledge, was not well studied in *P. multocida* and more experimental validation is desirable in the future.

Bacterial two-component regulatory systems and transcription factors are known to enable rapid adaptation to environmental conditions (Cases et al., 2003). We identified two genes *gabR* (OG0574) and *yofA* (OG1284) encoding HTH-type transcriptional regulators which showed significantly evidence for positive selection. Besides, the gene *qseC* (OG0958), which encodes a sensor kinase in the two-component QseBC quorum-sensing system, was also positively selected in *P. multocida*. Similar evidence for diversifying selection has been found in the PhoR/PhoB two component system in *Brucella* (Vishnu et al., 2015). These findings indicated positive selection of genes involved in transcriptional control may contribute to bacterial adaptation to various niches.

In summary, comparative genome analysis of 33 *P. multocida* strains revealed the pangenome compositions of *P. multocida*. Phylogeny of *P. multocida* was closely associated with serotyping rather than other factors like host origin or geographical location. Additionally, we performed a comprehensive analysis to investigate genetic content and diversification of virulence-associated genes in *P. multocida*. Our findings further indicated that evolutionary dynamics of pathogenic *P. multocida* were driven by positive Darwinian selection and homologous recombination. This study provides valuable targets for future researches on the mechanism of adaptive evolution and the host-pathogen interaction in *P. multocida*.

## Author contributions

Conceived and designed the experiment and wrote the paper: DG, ZX, and LQ. Performed the experiments: PC, DG, JL, and QJ. Contributed materials/analysis tools: DG and ZX. Analyzed the data: ZX, PC, and DG. All authors read and approved the final manuscript.

### Conflict of interest statement

The authors declare that the research was conducted in the absence of any commercial or financial relationships that could be construed as a potential conflict of interest.
