# Supplementary figures and images for: Genome-Wide Analyses Reveal Genes Subject to Positive Selection in Pasteurella multocida
Source: Front Microbiol. 2017 May 30;8:961. doi: 10.3389/fmicb.2017.00961 (PMC5447721; doi:10.3389/fmicb.2017.00961)

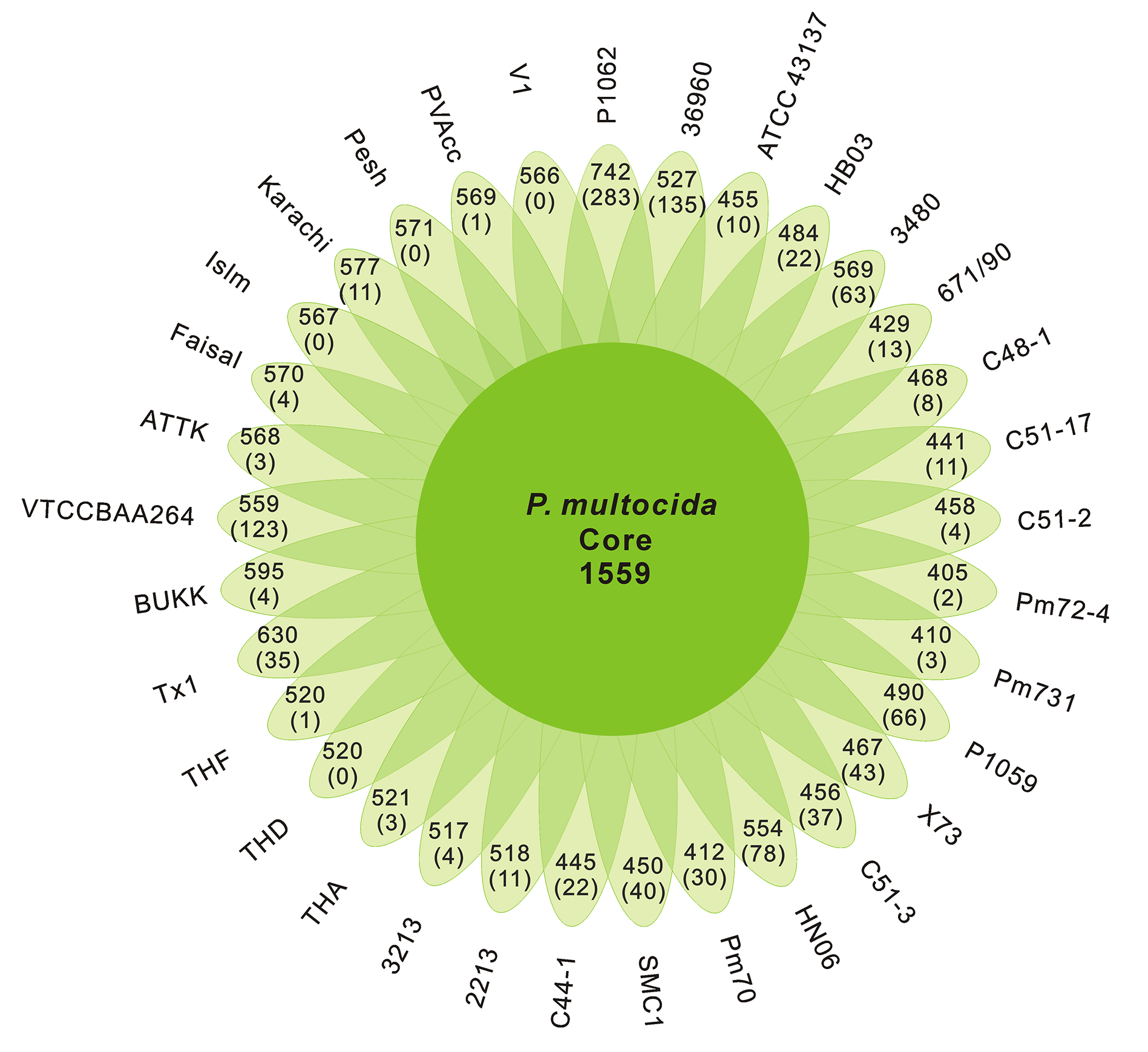

Supplement: Supplementary file 8 [file Image1.TIF]

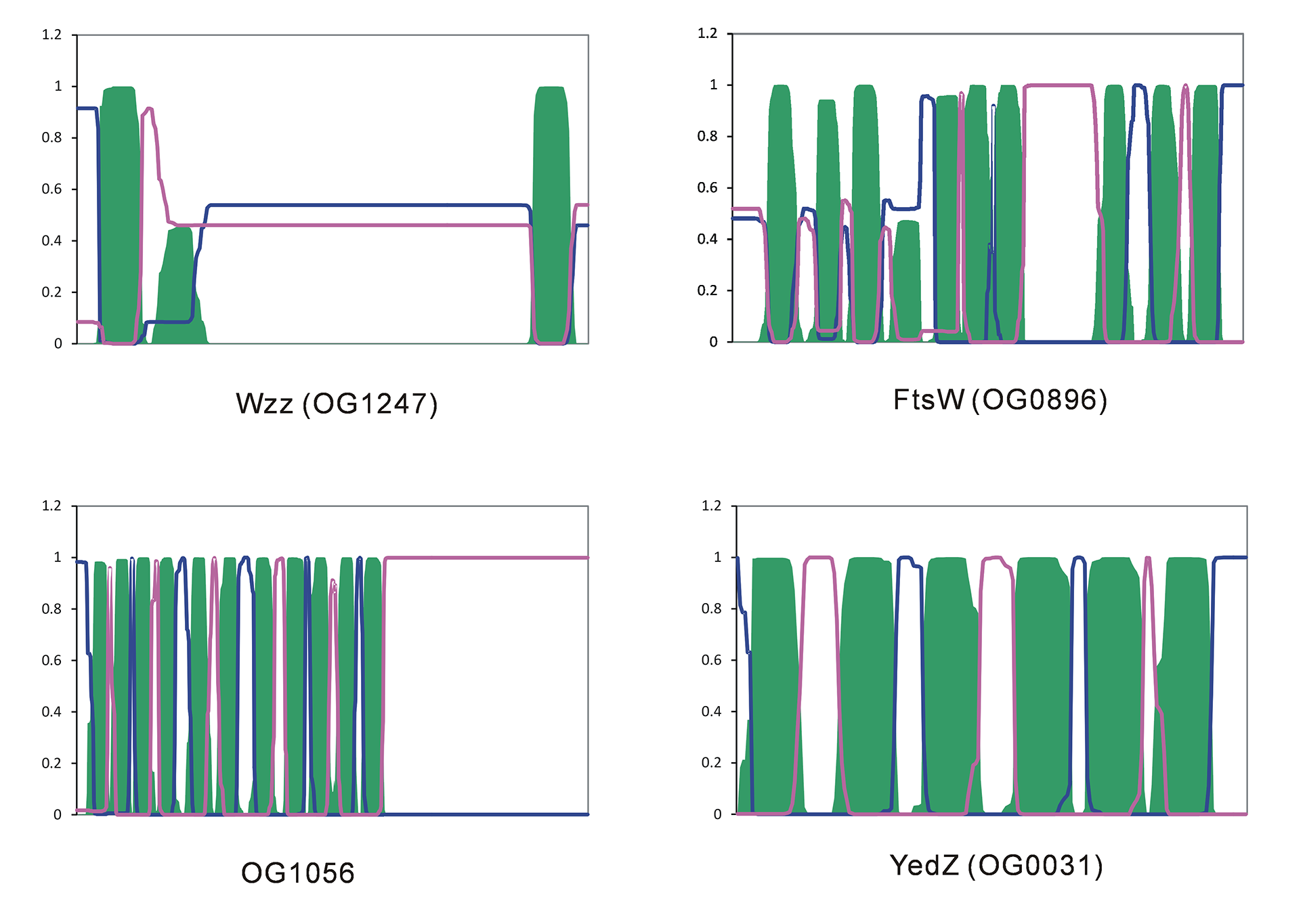

Supplement: Supplementary file 9 [file Image2.TIF]
